# Anesthetic management of a patient with dilated cardiomyopathy and purpura for interventional thrombectomy of both femoral artery: Case report

**DOI:** 10.1097/MD.0000000000037889

**Published:** 2024-05-10

**Authors:** Huazhen Wang, Yingming Zhu, Yongshan Nan, Xianglan Jin

**Affiliations:** a Department of Anesthesiology, Yanbian University, Yanbian University Hospital, Yanji, Jilin, P.R. China; b Department of Anesthesiology, Linyi Central Hospital, Linyi, Shandong P.R. China; c Department of Anesthesiology, Yanbian University Hospital, Yanji, Jilin, P.R. China.

**Keywords:** allergic reaction, anesthetic, dilated cardiomyopathy, interventional thrombectomy, purpura, thrombosis

## Abstract

**Rationale:**

Anesthesia management of patients with dilated cardiomyopathy (DCM) has always been a challenge for anesthesiologists. Eighty percent of patients with DCM have heart failure as the first symptom, which may be accompanied by arrhythmias, thromboembolism, etc. Thrombosis is a significant contributing factor to adverse cardiovascular and cerebrovascular events, and its risk is severely underestimated in the anesthetic management of DCM.

**Patient concerns:**

We present a case of a 54-year-old hypersensitive female patient with dilated cardiomyopathy and purpura who underwent an interventional thrombectomy under general anesthesia following a lower limb thromboembolism.

**Diagnosis:**

Patient underwent an interventional thrombectomy under general anesthesia, with in situ thrombosis occurring during the surgery.

**Interventions:**

After maintaining stable hemodynamics, proceed with the intervention to retrieve the embolus.

**Outcome:**

Patients in the advanced DCM developed acute thrombosis twice during embolization.

**Lessons:**

This case discusses the causes of intraoperative thrombosis and summarizes and reflects on the anesthesia management of this case, which has always been one of the difficult points for anesthesiologists to master. In the anesthesia management of DCM patients, it is also necessary to maintain hemodynamic stability, enhance perioperative coagulation management, use anticoagulants rationally, and avoid the occurrence of thrombotic events.

## 1. Introduction

Dilated cardiomyopathy (DCM) is primarily characterized by left ventricular, right ventricular, or bilateral ventricular enlargement and cardiac systolic dysfunction.^[1]^ It often leads to complications such as heart failure, arrhythmias, and thromboembolic events, and its perioperative mortality rate is high. In the present case, a hypersensitive patient with DCM and purpura had recurrent acute thrombosis during emergency interventional thrombectomy for acute bilateral lower extremity arterial embolism.

## 2. Case report

**Figure 1.**
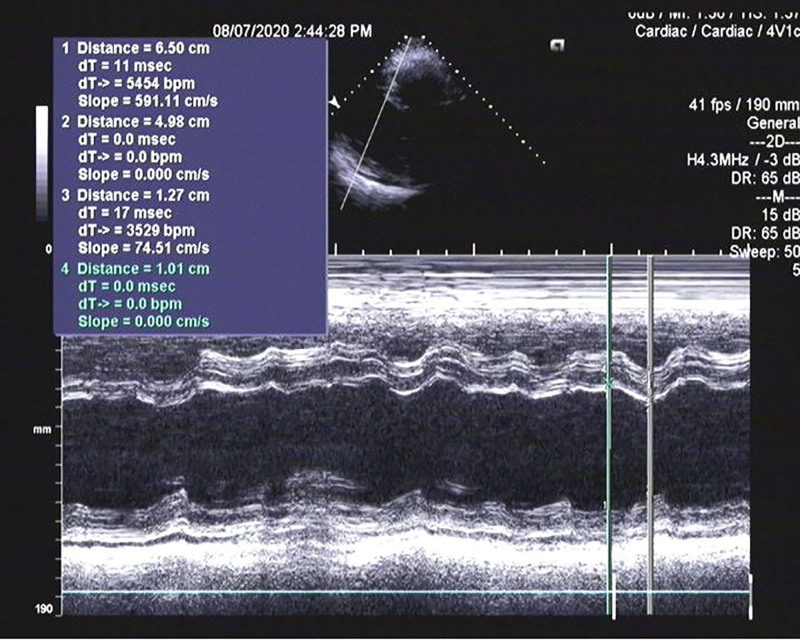
Echocardiogram showing a dilated and spherical left ventricle: Left ventricular end-diastolic diameter: 65 mm and left ventricular end-systolic diameter: 49.8 mm.

The patient is a 54-year-old female, 67 KG. She was admitted to the hospital on July 30, with “intermittent chest tightness and dyspnea for 10 years, aggravated for 2 days.” She had a history of hypertension for 10 years, with a maximum of 180/130 mm Hg, which was controlled by oral metoprolol; a history of diabetes mellitus for 6 years, which was controlled by oral metformin and glimepiride; a history of purpura for 10 years and eczema for 2 months. From 2010 to 2020, he was hospitalized several times for heart failure and was admitted to the cardiology department for further treatment because his condition worsened. On examination after admission: blood pressure was 100/80 mm Hg, clear speech, generalized skin rash, cyanosis of lips and mouth, bilateral jugular vein anger, clear breath sounds in both lungs, and a few wet rales were heard in the base of both lungs, apical beats were located at 0.5 cm lateral to the fifth intercostal space in the left midclavicular line, heart border was enlarged to the left, heart rat was 89/min, the rate was uniform, grade 3/6 was heard in the apical region, ventricular dilation is shown in Figure 1. A systolic murmur was heard in the apical region, the abdomen was flat and soft, and there was no sunken edema in both lower limbs. The ECG showed sinus rhythm, frequent ventricular asystole, incomplete left bundle branch block, ST-segment shift in leads V5-V6, and T-wave inversion; the chest CT showed an enlarged heart with a little chronic inflammation in the lower lobe of the left lung; Echocardiography showed a whole heart enlargement, diffusely reduced left ventricular wall motion, 30% left ventricular ejection fraction (LVEF), reduced left ventricular systolic and diastolic function, moderate mitral valve Inadequate closure, mild tricuspid regurgitation, mild pulmonary hypertension, small pericardial effusion, and no thrombus were observed. No abnormalities were seen in the 3 infarcts. The diagnosis was: “1, dilated cardiomyopathy (cardiac function class IV). 2, hypertension class 3 (very high risk). 3, type II diabetes mellitus. 4, arrhythmia (frequent ventricular asystole).” The cardiology department gave oral treatment with potassium chloride extended-release tablets 1 g thrice daily, spironolactone tablets 20 mg once daily, metoprolol extended-release tablets 11.875 mg once daily, and sacubitril valsartan 50 mg twice daily.

On the 4th day of admission, the patient was found to have pain in the left lower extremity with no palpable pulsation in the femoral arteries bilaterally, and the arteriovenous ultrasound of both lower extremities showed acute embolism in the arteries of both lower extremities, and the flow velocity of popliteal arteries was significantly reduced bilaterally, and no thrombosis was seen in the deep veins of both lower extremities. He was then transferred to the cardiovascular surgery department for further treatment.

After cardiovascular surgery evaluation, the patient had pain in both lower extremities for 4 hours, pale color of both lower extremities, low skin temperature, no depressed edema in both lower extremities, no palpation of the femoral, popliteal, posterior tibial, and dorsalis pedis arteries bilaterally, and no flow signal was detected by Doppler. The diagnosis of “acute bilateral lower extremity arterial embolism” was added, and the proposed procedure was performed on August 2, under emergency general anesthesia with balloon embolization of both lower extremity femoral arteries + drug-coated balloon dilation of both lower extremity arteries.

According to the clinical manifestations, the patient already belonged to the advanced stage of DCM, and all indicators suggested that the patient had a severe poor cardiac function and a high rate of perioperative cardiovascular event complications. The patient’s family was carefully explained the various anesthesia risks and complications and signed the anesthesia informed consent form. Considering the patient’s American Society of Anesthesiologists (ASA) class III–IV, general anesthesia with tracheal intubation was selected.

On August 2, at 18:00 the patient was admitted to the operating room with a blood pressure of 120/80 mm Hg, spontaneous respiration of 18/min, saturation of pulse oximetry (SpO_2_) of 93%, and a heart rate of 65/min. Under local anesthesia, the right internal jugular vein was cannulated, the right radial artery was punctured and invasive blood pressure (ABP) was monitored. Midazolam 2 mg, sufentanil 15 µg, cis-atracurium 15 mg, and etomidate 16 mg were given for induction of anesthesia. 6.5# reinforced tracheal tube was inserted orally under a visual laryngoscope at a depth of 22 cm, tidal volume 8ml/kg, respiratory rate 12/min. Blood pressure was controlled during induction by giving phenylephrine and nitroglycerin, and blood pressure was maintained at about 100–140/60–80 mm Hg during induction. Anesthesia was maintained with propofol 2 to 3 mg/(kg/h), dexmedetomidine 0.2 to 0.3 µg/(kg/h) pumped continuously with intermittent additional cis-atracurium. Intraoperative continuous pumping of norepinephrine 3 to 5 µg/(kg/min) and dopamine 6 to 8 µg/(kg/min) with the rate adjusted according to intraoperative ABP. Intraoperative monitoring of HR, ABP, end-breath carbon dioxide (PET CO_2_), SPO_2_. At 18:40, the operation started with embolization of the right femoral artery, and after removing the thrombus, the artery had good blood flow and the incision was sutured. At 20:30, the patient’s left femoral artery was treated in the same way, and at 22:00, it was decided to give a blood transfusion because the patient had 400 mL of bleeding and 3.6 mol/L of lactate. After starting plasma transfusion, blood pressure suddenly dropped, phenylephrine and ephedrine, and other vasoactive drugs were given, which were ineffective, and blood pressure was lowest at about 50/30 mm Hg. At 23:00, the left femoral artery was successfully embolized and balloon dilated, and the imaging showed good dilatation. At this time, the right femoral artery pulsation, no pulsation, so reopen the right original femoral artery incision, see thrombus formation, and carry out embolization once; August 3, 1:00 to explore the left femoral artery, also no pulsation, so reopen the left original femoral artery incision, see thrombus formation, and carry out embolization once again. The femoral artery pulsed well, and the operation was finished at 2:40 on August 3. The operation lasted for 8 hours, with 2000 mL of saline, 500 mL of hydroxyethyl starch, 1.5 µ of red blood cells, and 150 mL of plasma. 2500 mL of urine and 300 mL of bleeding during the operation.

## 3. Discussion

In the management of anesthesia for DCM, attention should be paid to avoiding fluid overload, preventing sudden increases in afterload, preventing the occurrence of arrhythmias or thromboembolic events, while maintaining adequate myocardial contractility and ejection fraction to meet the perfusion demands of vital organs, especially the coronary arteries. Due to surgical requirements, the patient needed systemic heparinization, which could lead to prolonged clotting time. However, the patient was in the late stages of DCM with severely reduced LVEF and congestive heart failure. The risk of ischemic perioperative stroke was higher when thrombosis occurred during endovascular thrombectomy.^[2]^ Fortunately, the patient only experienced in situ thrombosis without other adverse outcomes. This case highlights the importance of improving perioperative coagulation management in DCM patients to reduce the incidence of adverse events.

Thrombosis is a partial or complete blockage of the lumen of a blood vessel caused by the formation of an embolus in the bloodstream as a result of the activation of the coagulation system in physiological or pathological conditions. Damage to the vascular endothelium, altered physicochemical properties of the blood, and altered hemodynamics are the 3 main risk factors for thrombosis.^[3]^ This patient developed acute thrombosis twice during embolization. It may be because the patient was in advanced DCM, with severely reduced cardiac systolic function, and in a hypokinetic state, resulting in slow blood flow; severe intraoperative plasma allergic reaction, and vasodilation, resulting in a drop in blood pressure and dramatic hemodynamic fluctuations; Intraoperative bleeding is high, the effective circulating blood volume decreases, the hemoglobin content decreases, which makes the arterial blood flow rate decrease and platelet aggregation. It causes a decrease in oxygen-carrying capacity and insufficient tissue perfusion, which makes the tissue hypoxic, and hypoxia promotes the formation of thrombus,^[4]^ leading to acute thrombosis twice intraoperatively.

In this case, the patient was monitored only invasive blood pressure in addition to routine monitoring, and only these monitors were not enough. Central venous pressure, central venous saturation, stroke volume variation, EEG dual frequency index, and cardiac output and transesophageal echocardiography should also be performed. Cardiac output and transesophageal echocardiography monitoring.^[5]^ Better detection of hemodynamic changes and maintaining blood pressure close to preoperative baseline values during anesthesia management may help reduce the risk of perioperative stroke.^[2]^

Patients with DCM have severely decreased LVEF, congestive heart failure, and ventricular arrhythmias with a very fragile circulation. The choice of anesthetic drugs should avoid thiopental sodium, propofol, and inhalation anesthetics that are vasodilators and myocardial depressants. Choose drugs with low hemodynamic impact such as etomidate.^[6]^ The choice of anesthesia should be slow induction. Slow induction should be chosen to minimize the number of anesthetic drugs and hemodynamic fluctuations during induction, and small doses of dobutamine or phenylephrine can be pumped before induction to prevent the drop in blood pressure caused by vasodilatation during induction.^[7]^ During intubation and extubation, lidocaine or β-blockers can be given to control the heart rate and avoid tachycardia.^[5]^

The treatment did not sufficiently consider the patient’s history of purpura, failing to prevent possible allergic reactions during the procedure. Prophylactic use of steroids before surgery can reduce their incidence, avoid using hypersensitive anesthetics,^[8]^ and minimize the variety of anesthetic drugs used.

The process of anesthesia management must be comprehensive and meticulous. As an anesthesiologist, you cannot only focus on the indicators of various monitoring devices but also on the patient itself. The patient is hypersensitive and should be alert to changes in the patient’s skin, and conjunctiva to find allergic reactions in advance. Attention should also be paid to the patient’s fingers, mouth, lip color, skin temperature, and other conveniences, which can also provide certain information to determine the patient’s respiratory and circulatory status.

## 4. Summary

This case discusses the causes of intraoperative thrombosis and summarizes and reflects on the anesthesia management of this case, which has always been one of the difficult points for anesthesiologists to master. Better monitoring of vital signs can help us to detect and solve problems in a more timely manner. Through better preoperative evaluation, better preoperative preparation, and guiding our anesthesia management according to the anesthesia principles for DCM patients, we can avoid drastic hemodynamic fluctuations and prevent the formation of intraoperative thrombosis.

## Author contributions

**Project administration:** Yongshan Nan, Xianglan Jin.

**Supervision:** Yongshan Nan, Xianglan Jin.

**Writing – original draft:** Huazhen Wang.

**Writing – review & editing:** Huazhen Wang, Yingming Zhu.
